# Antiangiogenic activity of phthalides-enriched Angelica Sinensis extract by suppressing WSB-1/pVHL/HIF-1α/VEGF signaling in bladder cancer

**DOI:** 10.1038/s41598-017-05512-9

**Published:** 2017-07-14

**Authors:** Meng-Chuan Chen, Wen-Lin Hsu, Wen-Liang Chang, Tz-Chong Chou

**Affiliations:** 1 School of Dentistry, Graduated Institute of Dental Science, National Defense Medical Center, Taipei, Taiwan; 2 School of Medicine, Tzu Chi University, Hualien, Taiwan; 3 Department of Radiation Oncology, Buddhist Tzu Chi General Hospital, Hualien, Taiwan; 4 School of Pharmacy, National Defense Medical Center, Taipei, Taiwan; 5 Institute of Medical Sciences, Tzu Chi University, Hualien, Taiwan; 6 Department of Biotechnology, Asia University, Taichung, Taiwan; 7 China Medical University Hospital, China Medical University, Taichung, Taiwan

## Abstract

The hypoxia-inducible factor-1α (HIF-1α) plays a critical role in tumor angiogenesis. It has been reported that the acetone extract of Angelica sinensis (AE-AS) rich in phthalides is able to inhibit cancer cell proliferation. However, whether AE-AS reduces cancer angiogenesis remains unknown. In this study, we demonstrated that AE-AS significantly inhibited the angiogenesis *in vitro* and *in vivo* evidenced by attenuation of the tube formation in hypoxic human umbilical vascular endothelial cells (HUVECs), and the vasculature generation in Matrigel plug, the chicken chorioallantoic membrane, and tumors. Treatment with AE-AS markedly decreased the protein accumulation and transcriptional activity of HIF-1α, vascular endothelial growth factor (VEGF) expression/secretion, and VEGFR2 phosphorylation in hypoxic human bladder cancer (T24) cells and tumor tissues accompanied by a reduction of tumor growth. Notably, AE-AS-induced HIF-1α protein degradation may, at least partly, attribute to inhibition of WSB-1-dependent pVHL degradation. Moreover, VEGFR2-activated PI3K/AKT/mTOR signaling pathway in hypoxic T24 cells was greatly inhibited by AE-AS. Collectively, AE-AS may be a potential anticancer agent by attenuating cancer angiogenesis via suppression of WSB-1/pVHL/HIF-1α/VEGF/VEGFR2 cascade.

## Introduction

The intratumoral hypoxia (0.05–5% O_2_) is a common characteristic of most advanced solid tumors, leading to a resistance to radiotherapy and chemotherapeutic drugs, which causes a poor outcome of cancers due to induction of angiogenic and metastatic phenotypes^1, 2^. The hypoxia-stimulated hypoxia-inducible factor-1 (HIF-1) is a key transcriptional factor accounting for the hypoxic effects by upregulating glucose metabolism-related genes, such as glucose transporter 1 and carbonic anhydrase 9 to adapt the anaerobic metabolism in tumor, and its downstream pro-angiogenic genes, including vascular endothelial growth factor (VEGF) and VEGF-receptor-2 (VEGFR2), therefore promoting tumor angiogenesis and progression^3, 4^.

HIF-1 is a heterodimer composing of an inducible HIF-1α and a constitutive oxygen-insensitive HIF-1β/ARNT subunit. The biological functions of HIF-1 largely depend on the protein stability and activity of HIF-1α that is tightly controlled by oxygen tension^5^. The hypoxia-induced inactivation of prolyl-4-hydroxylases (PHDs), resulting in a decrease of the hydroxylation at proline residue (P402/P564) of HIF-1α, impairs the association of HIF-1α with von Hippel-lindau tumor suppressor (pVHL) and the degradation of HIF-1α, ultimately elevating HIF-1α protein accumulation. Then, HIF-1α translocates into nucleus, where it forms an active complex with HIF-1β to exert its transcriptional activity^6, 7^. Overexpression of HIF-1α is considered an important characteristic in several human solid tumors^8^. Conversely, suppressing HIF-1α expression and/or activity greatly reduces cancer angiogenesis, growth, and metastasis^9, 10^. Accordingly, attenuating HIF-1α-induced tumor angiogenesis is a potential strategy for cancer treatment.

The root of Angelica sinensis (RAS), known as Danggui in Chinese, is widely used to treat gynecological disorders, and it also acts as a common health food in many countries for a long time^11^. The RAS extracts have a variety of biological functions, including anticancer, neuroprotective, immunoregulatory, antioxidant, and hematopoietic activities^12^. Notably, different RAS extracts isolated by various solvents or methods have different pharmacological effects^13^. The acetone extract of RAS (AE-AS) has been reported to inhibit A549 human lung adenocarcinoma cell proliferation by inducing apoptosis^14^. However, whether AE-AS inhibits hypoxia-evoked cancer angiogenesis and the involvement of HIF-1α-regulated processes remain unknown. In the present study, we demonstrated that AE-AS is able to inhibit the angiogenesis in bladder cancer through suppressing HIF-1α induction and its-regulated pro-angiogenic signaling pathway.

## Results

### Chemical characteristics of AE-AS and the effects of AE-AS on reactive oxygen species (ROS) formation and HIF-1α expression/activity in hypoxic T24 cells

**Figure 1:**
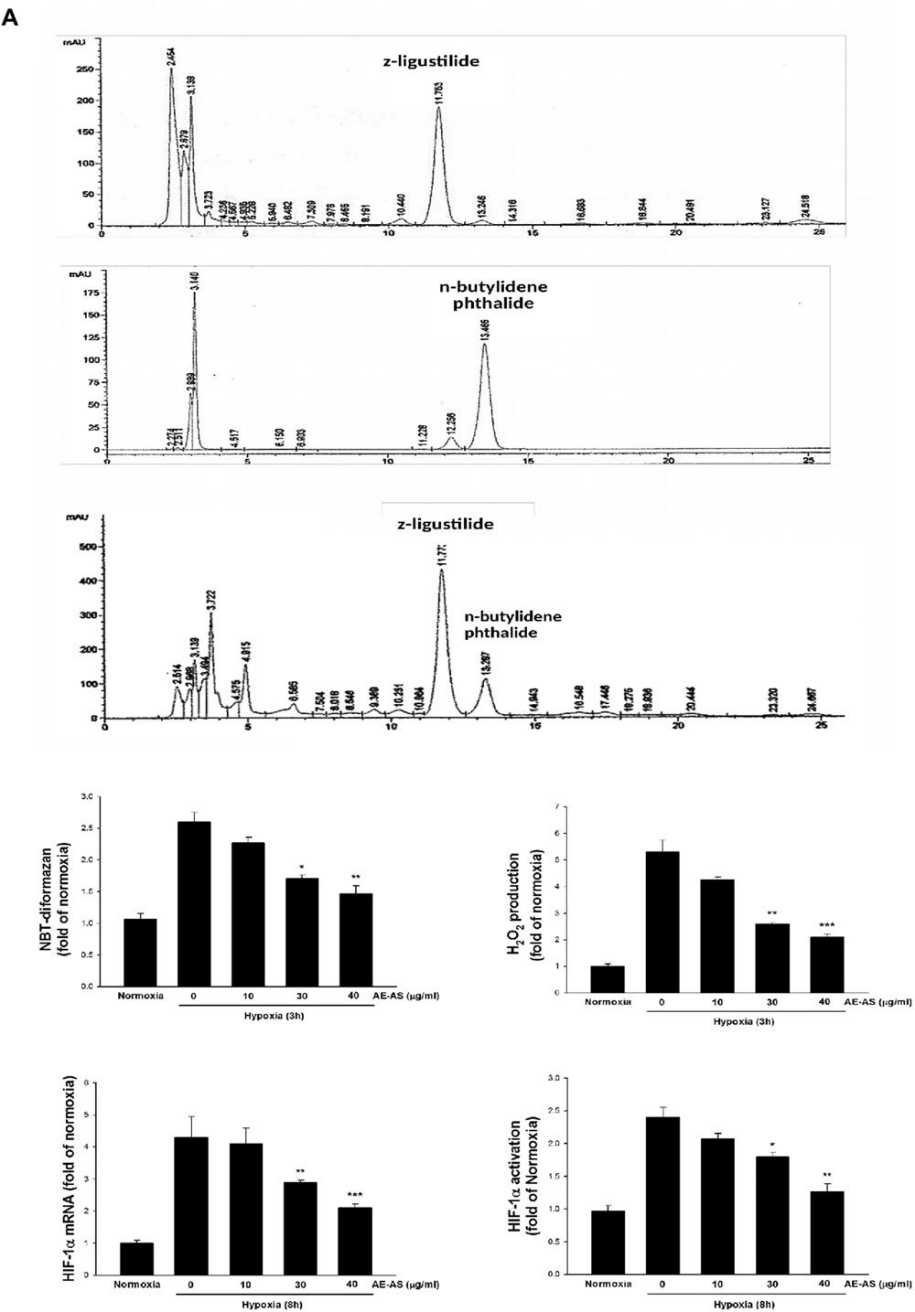
AE-AS inhibited ROS formation, HIF-1α mRNA expression and activation in hypoxic T24 cells. The analysis of chemical components of AE-AS extract (**A**), O_2_^−^ and H_2_O_2_ formation (**B**), HIF-1α mRNA expression (**C**), and HIF-1α transcriptional activity (**D**) were determined in various groups. Data was expressed as mean ± SEM (n = 5). *P < 0.05, **P < 0.01, ***P < 0.001 versus hypoxia-treated alone group.

**Figure 2:**
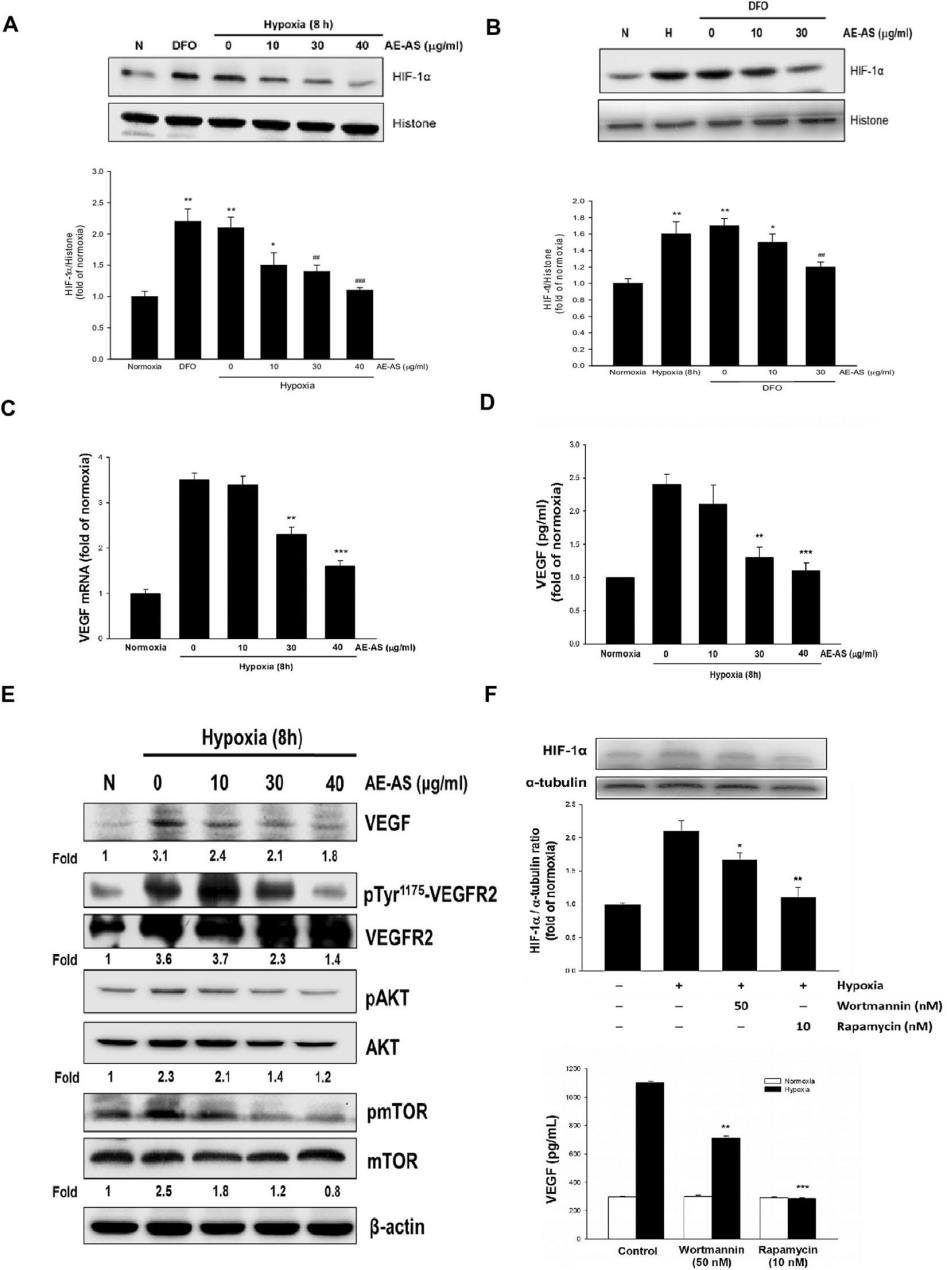
AE-AS reduced HIF-1α level, VEGF expression/secretion, and downstream signaling pathways in hypoxic T24 cells. The nuclear protein level of HIF-1α under hypoxia (**A**) or DFO treatment (**B**), VEGF mRNA (**C**), VEGF secretion (**D**), and related target gene expression (**E**) in various groups were analyzed. Data was expressed as mean ± SEM (n = 5). **P < 0.01, ***P < 0.001 versus normoxia-treated group, ^##^ *P* < 0.01, ^###^ *P* < 0.001 versus respective untreated group. The T24 cells were pretreated with rapamycin (10 nM) or wortmannin (50 nM) for 1 h followed by exposed to hypoxia for 8 h, and the HIF-1α protein expression and VEGF secretion were determined (F). *P < 0.05, **P < 0.01, ***P < 0.001 versus hypoxia-treated alone group.

The data obtained from HPLC analysis revealed that z-ligustilide and n-butylidenephthalide are major components accounting for 39% and 5% in the AE-AS, respectively (Fig. 1A). Hypoxia-induced ROS formation is known to increase HIF-1α protein stability^15^. Exposure of human bladder cancer (T24) cells to hypoxia for 3 h markedly increased O_2_^**−**^ and H_2_O_2_ formation, which was dose-dependently inhibited by AE-AS (Fig. 1B). Additionally, elevated HIF-1α mRNA expression (Fig. 1C), HIF-1α transcriptional activity (Fig. 1D), and nuclear HIF-1α protein level (Fig. 2A) in hypoxic T24 cells were significantly inhibited by AE-AS. Similarly, a marked increase of nuclear HIF-1α protein level in T24 cells caused by deferoxamine (DFO), an iron chelating agent acting as a hypoxia mimetic agent, was also inhibited by AE-AS (Fig. 2B).

### AE-AS inhibits VEGF expression/secretion and downstream signaling pathway in hypoxic T24 cells

The VEGF mRNA expression and VEGF secretion in hypoxic T24 cells were dose-dependently inhibited by AE-AS (Fig. 2C,D). In addition, hypoxia-induced VEGF protein expression, and the phosphorylation of VEGFR2 and AKT/ mammalian target of rapamycin (mTOR) cascade in T24 cells were reduced by AE-AS (Fig. 2E). To examine the role of PI3K/AKT and mTOR in the induction of HIF-1α and VEGF release, rapamycin, an inhibitor of mTOR, or wortmannin, an inhibitor of PI3K/AKT, was added. We found that blocking PI3K/AKT or mTOR activity dramatically attenuated hypoxia-induced HIF-1α expression and VEGF secretion (Fig. 2F), indicating that PI3K/AKT/mTOR signaling involves hypoxia-upregulated HIF-1α and VEGF.

### Effects of AE-AS on HIF-1α protein degradation and synthesis, as well as WSB-1 expression in hypoxic T24 cells

**Figure 3:**
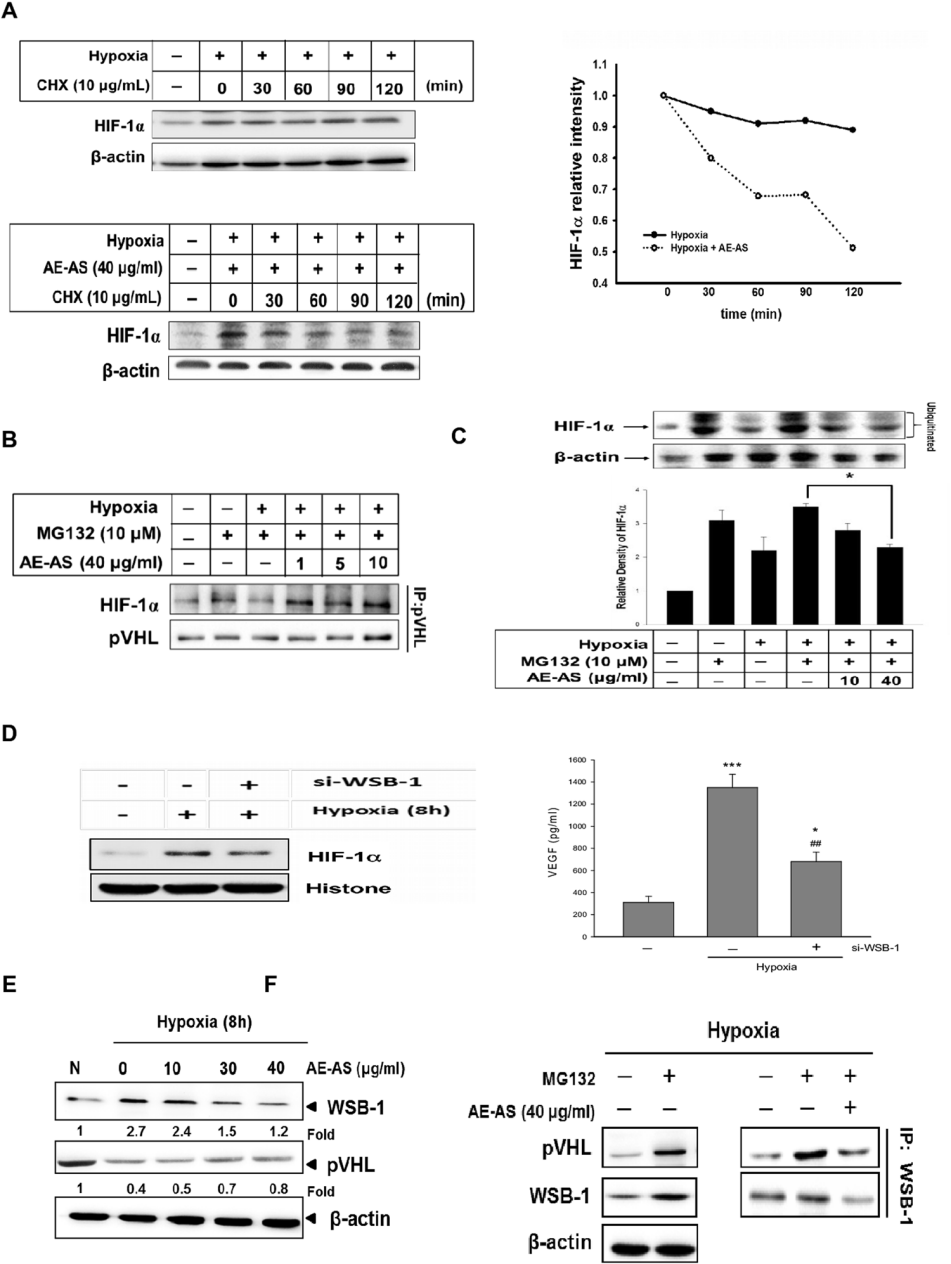
AE-AS decreased HIF-1α protein degradation and synthesis, and WSB-1-pVHL interaction in hypoxic T24 cells. T24 cells were exposed to hypoxia for 8 h followed by addition of cycloheximide (CHX, 10 μg/ml) in the presence or absence of AE-AS (40 μg/ml) for different time periods. The HIF-1α protein levels at indicated time were analyzed (**A**). In the presence of proteasome inhibitor MG132 (10 μM) and hypoxia, the interaction of pVHL and HIF-1α in various groups was examined (**B**). The T24 cells were pretreated with MG-132 for 1 h followed by exposure of hypoxia for 8 h in the presence or absence of AE-AS, and the HIF-1α protein levels were determined (**C**). Data was expressed as mean ± SEM (n = 5). *P < 0.05 versus hypoxia and MG132-treated alone cells. The nuclear protein level of HIF-1α and VEGF synthesis in the presence or absence of si-WSB-1 in hypoxic T24 cells were analyzed (**D**). The expression of WSB-1 and pVHL (**E**), and the association of WSB-1 with pVHL (**F**) in various groups were determined. *P < 0.05, ***P < 0.001 versus normoxic cells; ^##^ *P* < 0.01 versus hypoxia-treated alone cells.

After T24 cells exposed to hypoxia for 8 h, followed by addition of cycloheximide (CHX, 10 μg/mL) to block ongoing protein synthesis in the presence or absence of AE-AS (40 μg/mL), the relative protein levels of HIF-1α at different time periods were determined. Our results revealed that the estimated half-time of HIF-1α protein degradation in AE-AS-treated cells was about 115 min, which was much lower than the half time > 3 h in untreated hypoxic cells (Fig. 3A). In addition, decreased binding of HIF-1α to pVHL observed in hypoxic T24 cells evaluated by a co-immunoprecipitation (IP) assay was markedly reversed by AE-AS in the presence of MG132, a specific proteasome inhibitor (Fig. 3B), suggesting that enhanced HIF-1α degradation by AE-AS may be associated with activation of PHD-mediated HIF-1α hydroxylation and degradation. To further examine whether AE-AS affects HIF-1α protein synthesis, T24 cells were pretreated with MG132 for 30 min to prevent HIF-1α degradation, followed by hypoxia for 8 h in the presence or absence of AE-AS. As shown in Fig. 3C, hypoxia-induced newly synthesized HIF-1α protein was dose-dependently inhibited by AE-AS. Recent study has demonstrated that the WD repeat and SOCS box-containing protein-1 (WSB-1), a pVHL E3 ligase, can promote pVHL ubiquitination and proteasomal degradation in various tumors via WSB-1-pVHL interaction^15^. To further investigate whether WSB-1 regulates the HIF-1α/VEGF cascade in bladder cancer, the short interfering RNA (siRNA) WSB-1 was used. Consistently, hypoxia-induced nuclear HIF-1α level and VEGF synthesis in T24 cells was greatly reduced by si-WSB-1 (Fig. 3D), indicating that WSB-1 also stimulates the HIF-1α/VEGF cascade in bladder cancer. Moreover, the increased WSB-1 expression and decreased pVHL level occurred in hypoxic T24 cells were attenuated by AE-AS (Fig. 3E). Addition of MG132 dramatically increased pVHL protein level compared to hypoxia-treated alone cells, supporting that pVHL degradation is largely mediated by ubiquitin-proteasome system (UPS). Notably, in the presence of MG132, the interaction of WSB-1 and pVHL in hypoxic T24 cells was markedly inhibited by AE-AS (Fig. 3F). Collectively, AE-AS-mediated reduction of HIF-1α protein accumulation in hypoxic T24 cells may, at least partly, attribute to enhancing HIF-1α proteolysis via attenuation of WSB-1-dependent pVHL degradation and suppressing HIF-1α synthesis.

### AE-AS inhibits cancer cell migration and angiogenesis *in vitro* and *in vivo*

**Figure 4:**
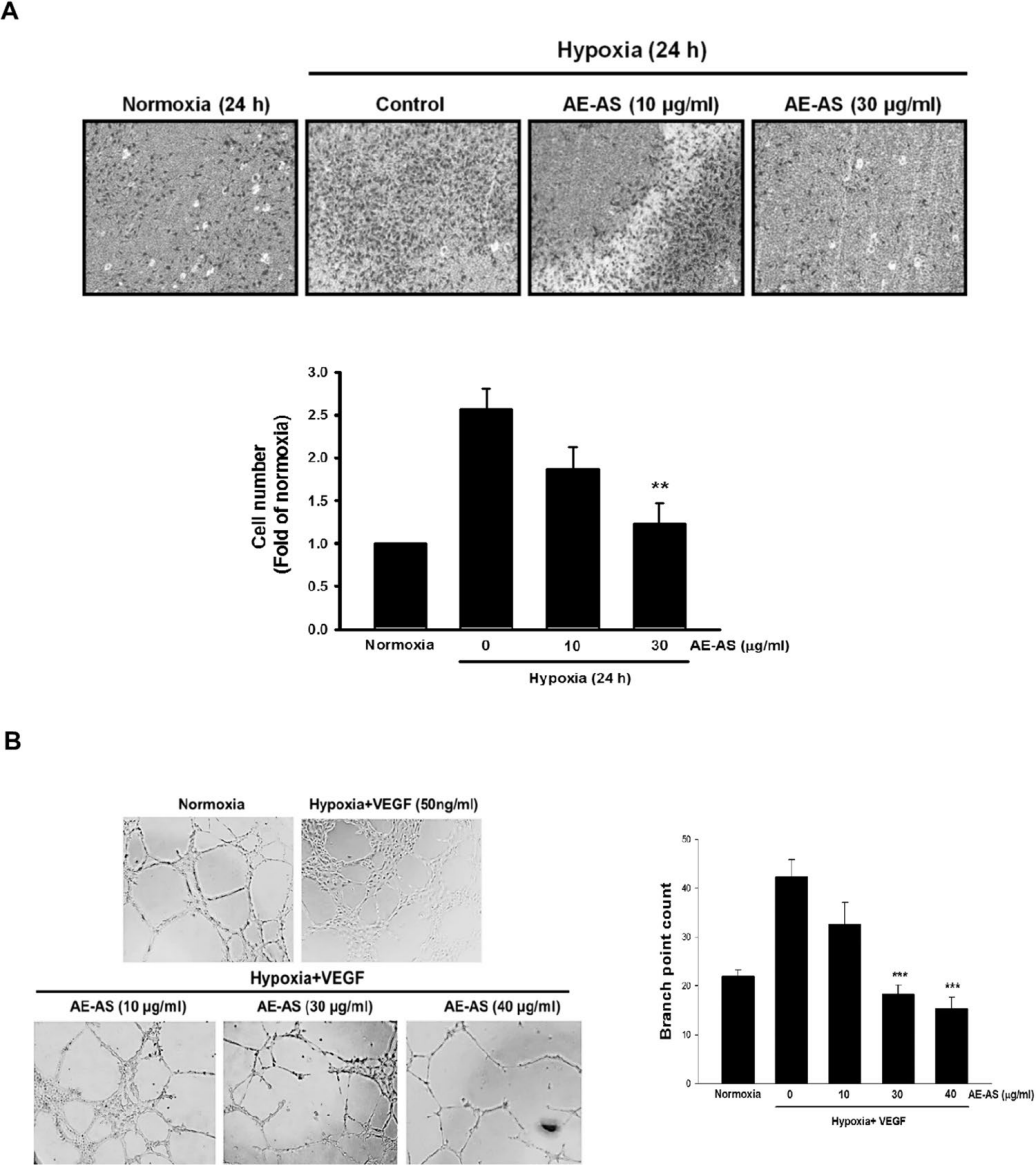
AE-AS inhibited the migration of T24 cells and the tube formation of HUVECs. The migration of hypoxic T24 cells (**A**) and the capillary-like tube formation in HUVECs in the presence of hypoxia and VEGF (**B**) in different groups were determined. Data was expressed as mean ± SEM (n = 5). In the migration tests: **P < 0.01 versus hypoxia-treated alone group. In the tube formation assay: ***P < 0.001 versus hypoxia + VEGF-treated alone group.

**Figure 5:**
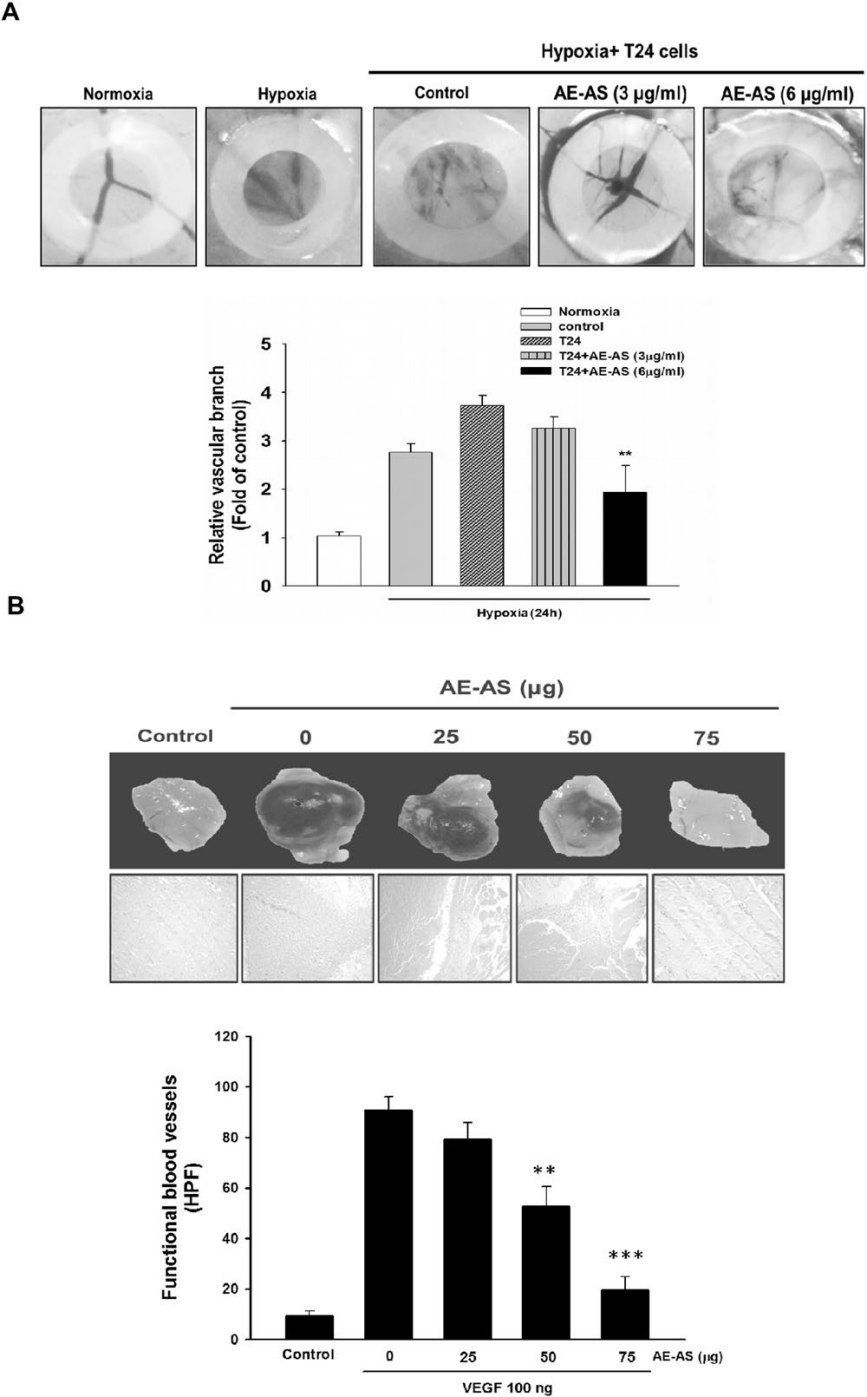
AE-AS inhibited the angiogenesis in CAM and Matrigel plugs. The vessel angiogenesis in CAM was determined and photographed, and the vascular branch was quantified by an angiogenesis-measuring software (**A**). Data was expressed as mean ± SEM (n = 5). **P < 0.01 versus hypoxia + T24-treated alone group. The functional vasculature formation induced by VEGF (100 ng) in Matrigel plugs was photographed and stained with H&E (200×). The infiltrating microvessels with intact RBC were quantified by manual counting using high power fields (200×) **P < 0.01, ***P < 0.001 versus VEGF-treated alone group.

Cancer cell migration is a crucial step for tumor angiogenesis and metastasis. Similarly, hypoxia-induced T24 cell migration was significantly reduced by AE-AS (Fig. 4A). To examine the antiangiogenic activity of AE-AS, different *in vitro* and *in vivo* angiogenesis models were used. Our results showed that hypoxia and VEGF-induced tube formation of human umbilical vascular endothelial cells (HUVECs) was dose-dependently inhibited by AE-AS (Fig. 4B). The hypoxia and T24 cell-stimulated angiogenesis in chicken chorioallantoic membrane (CAM) evidenced by an elevation of vascular branch was also attenuated by AE-AS (Fig. 5A). Consistently, VEGF-evoked functional vasculature formation in Matrigel plug seen in untreated mice was remarkably reduced by AE-AS treatment (Fig. 5B). These findings indicated that AE-AS exhibits an antiangiogenic activity *in vitro* and *in vivo*.

### AE-AS inhibits tumor growth and HIF-1α/VEGF/VEGFR pathway *in vivo*

**Figure 6:**
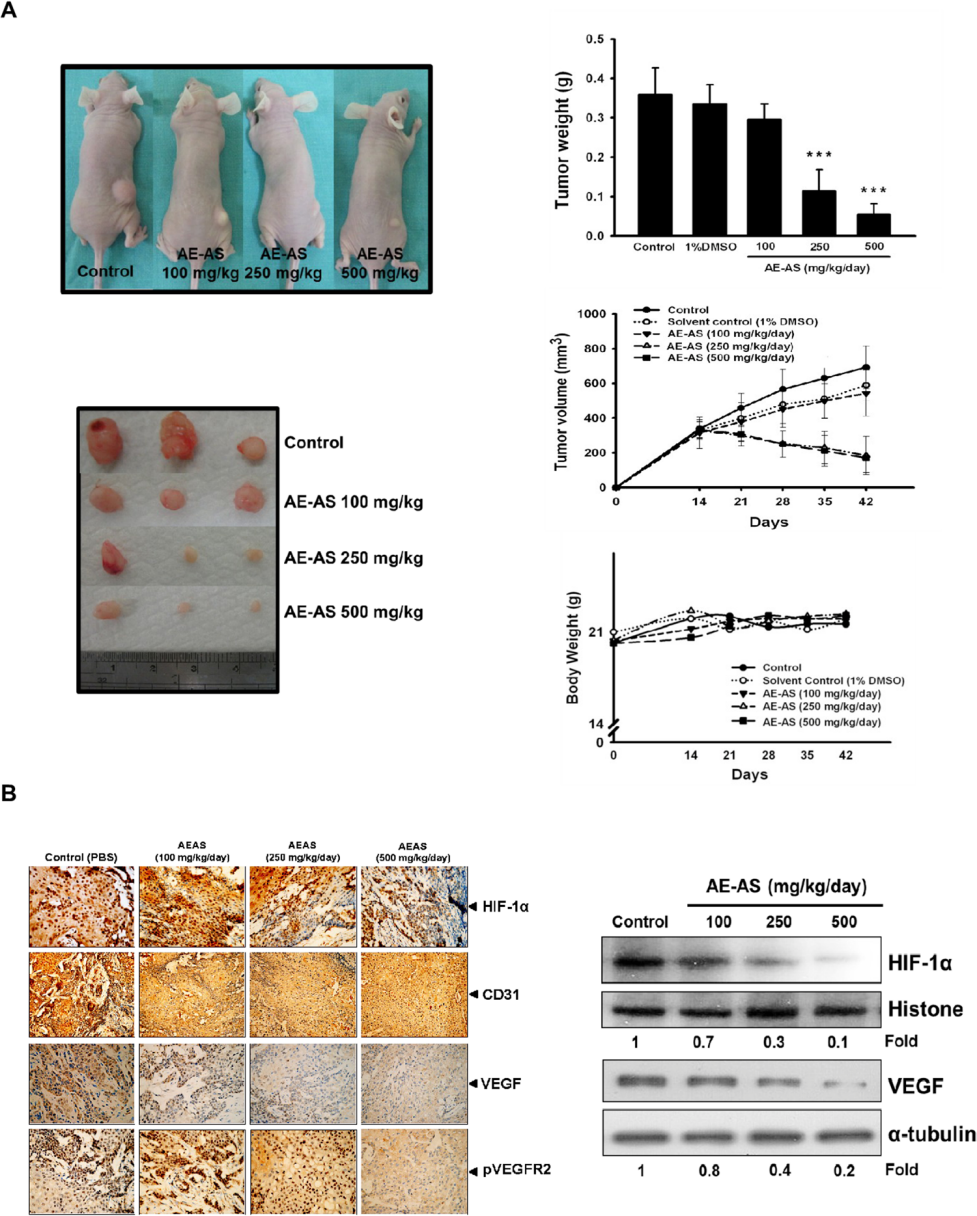
AE-AS reduced tumor angiogenesis and growth. The mice were injected with T24 cells (s.c.) for 15 days followed by treatment with different doses of AE-AS for 30 days, the images of tumor sections, tumor size and weight, and body weight of mice were measured (**A**). The protein expression of HIF-1α, VEGF, CD31 and pVEGFR2 in tumor tissues was determined by immunohistochemical staining or Western blotting assay (**B**). Data was expressed as mean ± SEM (n = 5). ***P < 0.001 versus untreated cancer mice (Control group).

In a xenograft mouse cancer model, the tumor size and weight of AE-AS-treated mice were much lower than that in untreated cancer mice (Control group), and AE-AS treatment did not affect the body weight of mice (Fig. 6A). As expected, the increased protein expression of HIF-1α, VEGF, pVEGFR2, and CD31 (PECAM-1), a specific endothelial cell marker, in tumor tissues determined by immunohistochemical staining or Western blotting assay was also greatly suppressed after AE-AS treatment (Fig. 6B).

## Discussion

A significant increase of the expression and activity of HIF-1α occurred in the tumor hypoxic microenvironment is a crucial force to trigger angiogenesis and tumorigenesis^4, 16^. It has been reported that the HIF-1α expression is highly correlated with the poor prognosis in cancer patients^8^. Therefore, inhibiting HIF-1α induction and its-evoked angiogenesis is a promising target for attenuating cancer progression. In this study, we demonstrated that AE-AS inhibits the angiogenesis in bladder cancer *in vitro* and *in vivo*, which may be associated with inhibition of HIF-1α-mediated responses.

The protein amount and biological functions of HIF-1α are controlled by multiple ways, including regulation of the protein synthesis, degradation, and transcriptional activity of HIF-1α, as well as its-regulated signaling pathways. Our data has confirmed that AE-AS inhibits the transcription of HIF-1α both at mRNA and protein levels in hypoxic T24 cells,. AE-AS also accelerated HIF-1α protein degradation with a less half-time compared to that of untreated hypoxic T24 cells (115 min vs. > 3 h). Therefore, inhibiting HIF-1α synthesis and enhancing HIF-1α protein degradation may contribute to AE-AS-mediated reduction of HIF-1α protein accumulation. Next, we explored the molecular mechanisms by which AE-AS inhibits HIF-1α protein stability and activity. The PHD is responsible for the hydroxylation and degradation of HIF-1α via UPS, and O_2_, 2-oxoglutarate and Fe^2+^ are required for PHD fully activity^17^. It is known that hypoxia-induced ROS production especially H_2_O_2_ can increase HIF-1α protein stability by oxidizing Fe^2+^ to Fe^3+^ via a Fenton reaction, causing a decrease of Fe^2+^ availability and PHD activity^18^. The factor-inhibiting HIF (FIH) is key enzyme for suppressing HIF-1α transcriptional activity by hindering the interaction of HIF-1α and the transcriptional co-activator p300/CBP^19^. Because the activity of FIH is dependent on the availability of O_2_, 2-oxoglutarate, and Fe^2+^, overproduction of H_2_O_2_ under hypoxic condition also acts a negative regulator for FIH activity^20, 21^. Accordingly, AE-AS-mediated inhibition of ROS production in hypoxic T24 cells may be a crucial mechanism for promoting HIF-1α degradation, and suppressing the transcriptional activity of HIF-1α by preventing PHD and FIH inactivation.

It has been reported that loss or mutation of pVHL remarkably enhances HIF-1α protein accumulation, and tumor angiogenesis, proliferation and metastasis^22–24^, supportting that preventing pVHL protein loss is capable of attenuating tumorigenesis. Recent study has identified that WSB-1 reduces the pVHL protein amount by stimuating pVHL ubiquitination and proteasomal degradation, thereby stabilizing HIF-1α^15^. Importantly, WSB-1 is a target gene of HIF-1α^24^, suggesting a positively feedback loop existing in WSB-1 and HIF-1α via regulation of pVHL turnover. Our data showed that knocking down WSB-1 with si-WSB-1 markedly reversed the elevation of nuclear HIF-1α level and VEGF synthesis in hypoxic T24 cells, indicating that WSB-1 plays a crucial role in the activation of HIF-1α/VEGF cascade in bladder cancer. To date, whether AE-AS affects the linkage of WSB-1 with pVHL is still unknown. It is the first study to confirm that AE-AS attenuated the WSB-1 induction accompanied by a decrease of pVHL protein amount in hypoxic T24 cells. The interaction of WSB-1 and pVHL was also inhibited by AE-AS, which may be due to downregulation of WSB-1. Therefore, the mechanism underlying AE-AS-mediated attenuation of HIF-1α protein stability may, at least partly, attribute to suppressing WSB-1-dependent pVHL degradation.

Upon activation of HIF-1α, an elevation of VEGF synthesis and secretion is essential for cancer angiogenesis and progression^25^. Once VEGF binding to its receptors particularly VEGFR2 results in an autophosphorylation (activation) of VEGFR2 and activation of PI3K/AKT/mTOR pathway^26–28^. Blocking PI3K/AKT or mTOR activity dramatically diminished hypoxia-induced HIF-1α expression and VEGF generation in hypoxic T24 cells, suggesting that the VEGF/VEGFR2/PI3K/AKT/mTOR cascade contributes to the induction of HIF-1α and VEGF. Of note, hypoxia-stimulated VEGF transcription/secretion, VEGFR2 phosphorylation, and AKT/mTOR pathway in T24 cells were markedly inhibited by AE-AS. Collectively, the inhibitory effect of AE-AS on HIF-1α-induced angiogenesis may be mediated by multiple mechanisms, including suppressing the transcriptional activity, protein synthesis and stability of HIF-1α, as well as impairing VEGFR2-activated pro-angiogenic signaling. Consistent with the findings *in vitro*, administration of AE-AS greatly reduced the protein expression of HIF-1α, VEGF, and pVEGFR2, as well as tumor angiogenesis reflected by a decrease of CD31 expression. Accumulating evidence has demonstrated that tumor growth is angiogenesis dependent^29^. As expected, treatment with AE-AS significantly attenuated the tumor size without reducing the body weight of mice, which confirms that AE-AS has an anticancer activity and the dose of AE-AS (100–500 mg/kg/day) used in this study is safe or low toxicity for mice.

Angelica sinensis (AS) contains different types of bioactive compounds, such as phthalides and polysaccharide^30^. It has been reported that z-ligustilide and n-butylidenephthalide, the major phthalides in RAS, can be extracted with various organic solvents, such as acetone. Previous study has demonstrated that the phthalides possess anti-proliferative and cytotoxic effects in colon cancer cells (HT-29)^31^, and the n-butylidenephthalide is able to inhibit the angiogenesis of HUVECs^32^. Conversely, the aqueous extracts of AS rich in polysaccharide (60%) can promote the angiogenesis in zebrafish^33^. These results indicate that different anti- or pro-angiogenic components may present in specific extracts of AS, which may be a possible reason accounting for the contrasting effects of different AS extracts on angiogenesis. The analysis of the chemical composition revealed that there are two major constituents: z-ligustilide (39%) and n-butylidenephthalide (5%) in the AE-AS extract. Additionally, AE-AS, z-ligustilide, and n-butylidenephthalide have a similar activity on the inhibition of HIF-1α and VEGF expression in various bladder cancer cell lines, such as T24, HT1376, and HT1197 (supplementary data). Thus, the antiangiogenic activity of AE-AS may be largely mediated by the actions of z-ligustilide, the most abundance in the extract. It has been reported that there are some minor z-ligustilide dimers including E-232 existing in the roots of Angelica sinensis. However, these z-ligustilide dimers can be easily decomposed into z-ligustilide in hot condition^34^, suggesting that the z-ligustilide dimers are heat-instable. Because the heating system was also used in the extraction and concentration procedure of AEAS, the z-ligustilide dimer E-232 may be decomposed into z-ligustilide and it was not detected in the AE-AS extract. Accordingly, the z-ligustilide dimers may be not involved in the actions of AE-AS. Notably, it has been demonstrated that Z-ligustilide exhibits an estrogenic activity. Combined treatment with Z-ligustilide significantly restored the anticancer activity of tamoxifen in estrogen receptor α negative (ERα^−^) breast cancer cells by activating the protein expression and transcriptional activity of ERα^35^. Whether the estrogenic activity of AE-AS contributes to the antiangiogenic effect in bladder cancer needs further investigations.

Previous pharmacokinetic studies have reported that oral z-ligustilide and butylidenephthalide have a low bioavailability with 2.6% and 19%, respectively, due to their extensive first-pass intestinal and hepatic metabolism^36, 37^. Interestingly, in the body, z-ligustilide can be metabolically converted to butylidenephthalide. When the administration of the two bioactive components is combined with AS extracts or fractions, the pharmacokinetic profiles are markedly altered^37^, implying that there are mutual conversion/interactions among these components existing in AS. This highlights the importance for identifying the distinct bioactive ingredients and the pharmacokinetic interactions between components in various AS extracts to ensure their proper use especially in angiogenesis-related diseases. In conclusion, we demonstrated that AE-AS exhibits a potent antiangiogenic activity in bladder cancer through suppressing WSB-1-induced pVHL degradation, HIF-1α induction/activity, and angiogenesis-related signaling pathways. Taken together, AE-AS may be a potential anticancer agent by inhibiting cancer angiogenesis.

## Methods

### Materials

The antibodies including anti-HIF-1α, anti-VEGF, anti-CD31 (PECAM-1), anti-VHL, anti-WSB-1, anti-β-actin, anti-α-tubulin and fluorescein isothiocyanate (FITC)-coupled secondary antibody were purchased from Santa Cruz Biotechnology (Santa Cruz, CA, USA). The anti-VEGFR2, anti-phospho-VEGFR2, anti-AKT, anti-phospho-AKT, anti-mTOR, and anti-phospho-mTOR were purchased from Cell Signaling Technology (Danvers, MA, USA). Horseradish peroxidase (HRP)-labeled secondary antibody and goat anti-rabbit IgG-biotin secondary antibody were obtained from Abcam (Cambridge, MA, USA). The z-ligustilide, n-butylidenephthalide, and other chemical reagents used in this study were purchased from Sigma (St. Louis, MO, USA)

### Preparation of extracts from angelica sinensis

The roots of Angelica sinensis (Oliv.) Diels were provided and extracted by Dr. Wen-Liang Chang. The dried and powdered roots of A. sinensis were extracted with acetone and concentrated under reduced pressure to yield an acetone extract (AE-AS) as previously described^14^. The AE-AS was dissolved in DMSO for subsequent tests.

### Determination of the chemical ingredients of AE-AS

For chemical composition analysis of AE-AS, a reverse phase HPLC system was used (Agilent 1100, Cosmosil column, 5C18-AR-II Waters, 5 μm, 4.6 mm x 250 mm) under the assay condition: a mobile phase consisting of 70% MeOH/30% H_2_O at a flow rate of 1.0 ml/min with an injection volume of 20 μl and an UV detection wavelength of 270 nm.

### Cell culture and hypoxic treatment

The T24 and HUVECs were purchased from the Bioresource Collection and Research Center (Taipei, Taiwan). T24 cells were incubated in RPMI1640 supplemented with 10% fetal bovine serum (Thermo Fisher Scientific Inc., Waltham, Utah, USA), 2 mM L-glutamine, and 100 U/ml penicillin-streptomycin (Gibco, Carlsbad, St. CA, USA). HUVECs were grown in M199 containing 10% FBS, endothelial cell growth supplement (ECGS, 0.03 mg/mL) and kanamycin (50 U/ml) purchased from Sigma (St. Louis, USA). For hypoxic exposure, cells were incubated in serum starved medium for 24 h, followed by placing in a sealed hypoxic chamber flushed with a gas mixture of 94% N_2_, 5% CO_2_ and 1% O_2_.

### Reactive oxygen species (ROS) measurement

To measure superoxide anion (O^2−^) formation, nitroblue tetrazolium (NBT, 1 mg/mL PBS, pH = 7.2) was added into T24 cells (2.5x10^5^ cell/well) for 30 minutes, and the absorbance at 560 nm was determined. The intracellular H_2_O_2_ was measured by the change of fluorescence resulting from oxidation of 2’,7’-dichlorofluorescein diacetate (H_2_DCF-DA) (Invitrogen Molecular Probes, Carlsbad, CA, USA).

### Quantitative real-time PCR assay

Total RNA was isolated from cells by using TRIzol reagent (Life Technologies, Carlsbad, CA, USA). Briefly, 3 μg of total RNA, 1 μl of primers, and 5 μl of Power SYBR Green PCR Master Mix (Applied Biosystems, Carlsbad, CA, USA) were incubated in 10 μl at 50 °C for 10 min and 95 °Cfor 5 min, followed by 45 cycles of 95 °C for 10 s, and 72 °C for 30 s. Each reaction was run in duplicate and the threshold (*C*_T_) values for each mRNA were subtracted from that of β-actin mRNA, averaged, and converted from log-linear to linear term.

The primer sequences used in the PCRs were as follows:

HIF-1α (forward) 5′-TGAGGAAATGAGAGAAATGCTTACA-3′

HIF-1α (reverse) 5′-ACACTGAGGTTGGTTACTGTTGGT-3′

VEGF (forward) 5′-CCAGCACATAGGAGAGATGAGCTT-3′

VEGF (reverse) 5′-TCTTTCTTTGGTCTGCATTCACAT-3′

β-actin (forward) 5′-TCTGGCACCACACCTTCTACAA-3′

β-actin (reverse) 5′-GTACATGGCTGGGGTGTTGAAG-3′

### HIF-1α activity and VEGF measurement

The HIF-1α activity was determined by HIF-1α Combo Transcription Factor Assay Kit according to the manufacturer’s instructions (Cayman, Ann Arbor, MI, USA). The amounts of VEGF were measured by using VEGF enzyme-linked immunosorbent assay (ELISA) kit (R&D Systems, MN, USA).

### Western blotting assay

The nuclear and cytosolic extracts of cells (1x10^6^ cell/10 cm dish) were prepared by using NE-PER nuclear and cytoplasmic extraction reagents (Thermo Fisher Scientific Inc., Waltham, Utah, USA). The protein samples (30–100 μg) were separated on a 9% SDS-PAGE, and transferred onto nitrocellulose membranes. After blocking with 5% nonfat dry milk in 5% TBST for 1 h, the membranes were incubated with various appropriately diluted primary antibody of target genes for overnight at 4 °C. After washing with TBST, the membranes were incubated with horseradish peroxidase-conjugated secondary antibody for 1 h and the immunoreactivity was visualized by using enhanced HRP substrate luminol reagent (Milipore, Billerica, MA, USA).

### Short interfering (si) RNA transfection

The WSB-1 siRNAs and control siRNAs were purchased from Santa Cruz Biotechnology (CA, USA). Cells were grown to 70% confluence, and siRNAs are transfected by using the lipofectamine RNAiMAX reagent according to the manufacturer’s instructions (Santa Cruz Biotechnology). The final concentration of siRNA for transfection was 10 nM.

### Co-immunoprecipitation assay

The cell lysates (1 mg) of cells were incubated with 1 μg of anti-rabbit WSB-1 antibody (Santa Cruz Biotechnology) in a total volume of 300 μl of ice-cold lysis buffer containing 50 mM Tris-Cl (pH = 7.5), 150 mM NaCl, 1% Nonidet P-40, 10% glycerol, and protease inhibitor cocktail containing 1 mM DTT, 1 mM EDTA, and 1 mM PMSF. After rocking for 24 h at 4 °C, 60 μl of Protein A magnetic beads (Millipore Corporation, Billerica, MA, USA) was added and incubated for overnight at 4 °C followed by washing four times with lysis buffer. The precipitates were boiled at 95 °C for 10 min. The eluted proteins were separated on 9% SDS-polyacrylamide gel and the target genes were detected by Western blot analysis with anti-WSB-1 (1:200 dilution) or anti-human VHL (1:200 dilution).

### Cell migration assay

The migration assay was performed using a 24-well Boyden chamber with 8 μm pore size polycarbonate membrane (Millipore, Boston, MA, USA). Briefly, T24 cells (5 × 10^4^ cells/well) were added into the chamber containing 200 μl of serum-free medium, and 750 μl RPMI 1640 with 10% FBS was added into the lower compartment. After incubation in a hypoxic chamber for 24 h, the T24 cells remaining in the upper chamber were removed with cotton swabs. The cells on the lower surface of the membrane were fixed in 95% ethanol and stained with 0.1% crystal violet. The number of migrated cell was counted under a light microscope.

### Capillary-like tube formation assay

Matrigel (12.5 mg/ml, BD Biosciences, Bedford, MA, USA) was thawed at 4 °C for overnight, and 50 μl Matrigel was quickly pipetted onto 96-well plate and allowed to solidify for 10 min followed by addition of HUVECs (1 × 10^4^ cells/well). After adhesion of the cells, the medium was removed and replaced by fresh medium supplemented with VEGF (50 ng/ml) and indicated concentrations of AE-AS, followed by an incubation for 18 h under hypoxic condition. The tube formation was photographed with an Olympus IX 70 invert microscope (Olympus America, Inc., Melville, NY, USA), and the vessel branches were quantified with an angiogenesis-measuring software (KURABO, Osaka, Japan).

### Chicken chorioallantoic membrane (CAM) assay

The pathogen-free (SPF) fertilized white Leghorn chicken eggs purchased from Animal Health Research Institute (Taipei, Taiwan) were maintained at 37 °C under constant humidity. At day 8, eggs were candled and windows were opened on the shell to expose the CAM. Then, AE-AS was injected into the ring on CAM followed by 24 h incubation in the presence of hypoxia and T24 cells (2 × 10^4^/20 µl). The blood vessel branches on CAM were quantified with an angiogenesis-measuring software (KURABO, Osaka, Japan).

### Matrigel plug angiogenesis assay

Matrigel (0.5 ml/plug) containing 100 ng VEGF and 20 units heparin without or with AE-AS in a liquid form at 4 °C was injected in the midventral abdominal region of 5–6 week-old C57BL/6 mice for 7 days. The intact Matrigel plugs were removed and stained by hematoxylin and eosin (H&E) to identify the formation of new microvessels. The number of functional microvessels filled with erythrocytes was counted manually using a microscope in high power field (HPF; 200 × ).

#### Xenograft mouse model

The 7-week-old female athymic nude mice (BALB/c) weighing ~25 g were used. After subcutaneous injection of T24 cells (2 × 10^6^ cells per mouse) for 15 days and the tumors reached a palpable size, the mice were administrated with vehicle (distilled H_2_O) or AE-AS (100–500 mg/kg/day, p.o.) for 30 days. The body weight, tumor weight, and tumor size were determined by a caliper following the formula of V = lw^2^/2, wherein l is the length (mm) and w is the width (mm) diameter of tumor. The animal care and experimental procedures were conducted in accordance with the Guiding Principle in the Care and Use of Animals and approved by Animal Care and Use Committee of National Defense Medical Center, Taipei, Taiwan (IACUC 12156).

### Immunohistochemical staining

The tissues were fixed with 10% formaldehyde and embedded in paraffin followed by incubation of target primary antibody for overnight and goat anti-rabbit IgG-biotin secondary antibody (1:300, Abcam) for 1 h. After extensive washings with PBS, the samples were stained with diaminobenzidine peroxidase substrate and photographed.

### Statistical analysis

The experimental data were expressed as the mean ± SEM of five independent experiments. One-way ANOVA with post hoc Bonferroni test was used for statistical analysis. Results were considered significant difference at a value of *P* < 0.05.

## Electronic supplementary material

Supplementary information
