# Supplementary material for: Antiangiogenic activity of phthalides-enriched Angelica Sinensis extract by suppressing WSB-1/pVHL/HIF-1α/VEGF signaling in bladder cancer
Source: Sci Rep. 2017 Jul 14;7:5376. doi: 10.1038/s41598-017-05512-9 (PMC5511260; doi:10.1038/s41598-017-05512-9)
Supplement: Supplementary file 1 — Supplementary information [file 41598_2017_5512_MOESM1_ESM.pdf]

Antiangiogenic activity of phthalides-enriched Angelica Sinensis extract by suppressing WSB-1/pVHL/HIF-1α/VEGF signaling in bladder cancer

Meng-Chuan Chen[1,†], Wen-Lin Hsu[2,†], Wen-Liang Chang[3], Tz-Chong Chou[4,*]

Correspondence to Tz-Chong Chou, Ph.D.

Institute of Medical Sciences, Tzu Chi University, 6F, Xie-Li Building, No. 707, Sec. 3, Zhongyang Rd., Hualien, 97002, Taiwan. Tel: 886-3-8561825 ext. 5620; Fax: 886-3-8573710; E-mail: chou195966@gmail.com

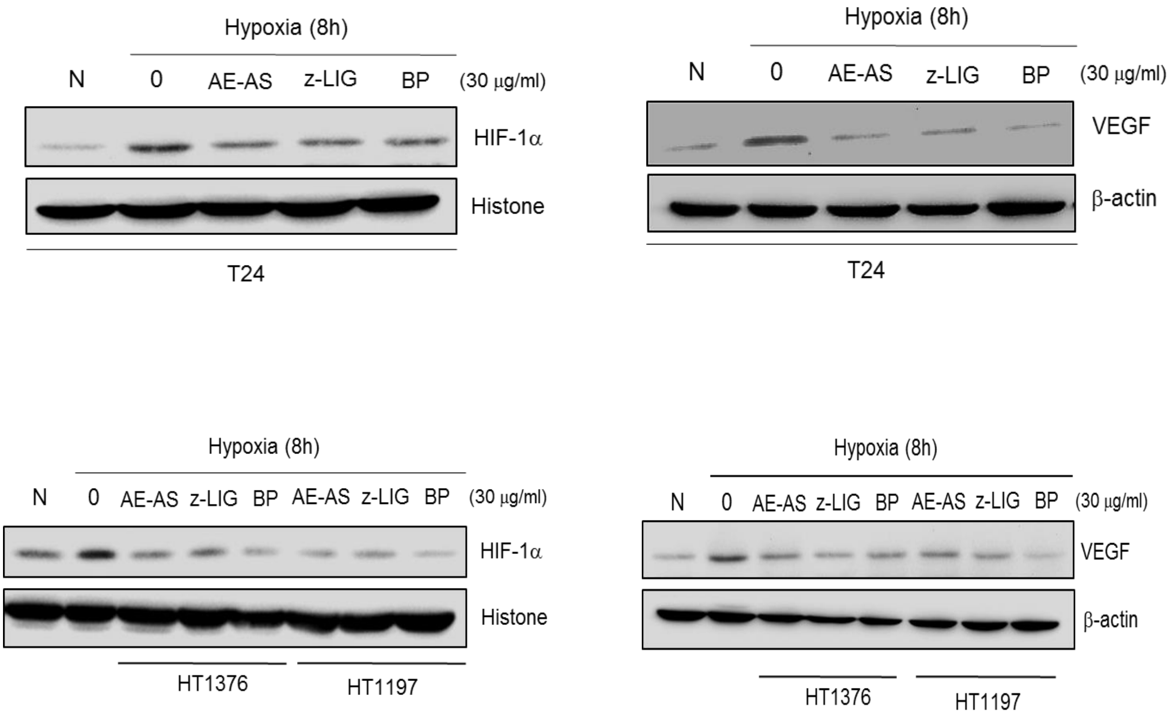

Effects of AE-AS, z-ligustilide (z-LIG) and n-butylidenephthalide (BP) on hypoxia-induced the expression of HIF-1α and VEGF in various bladder cancer cell lines.

Supplementary figure 1

Supplementary information of original blots

Figure 2A.

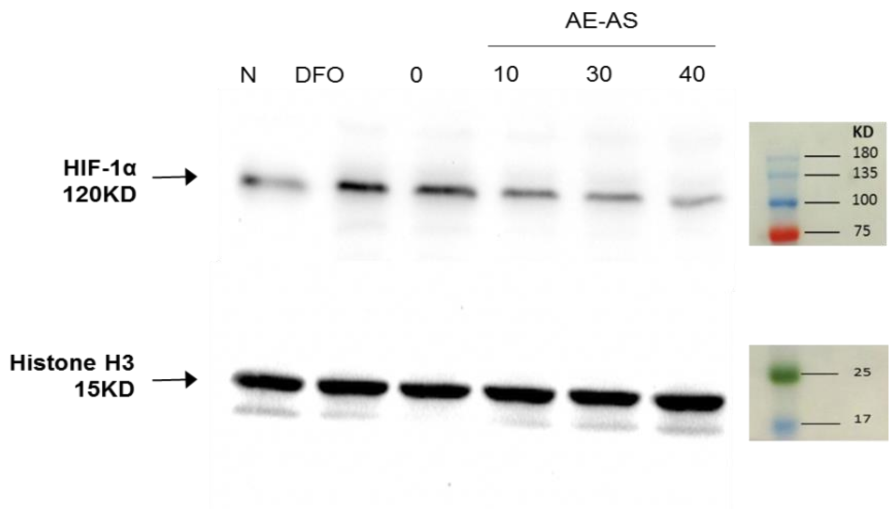

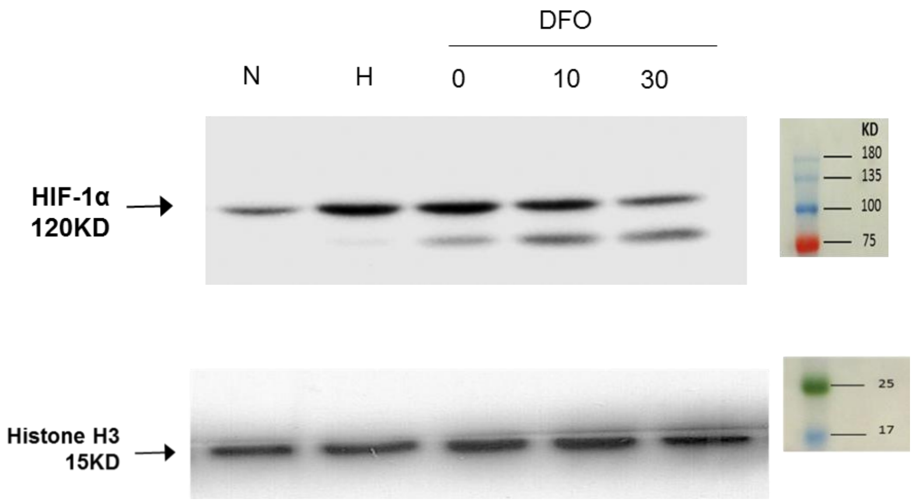

Figure 2E.

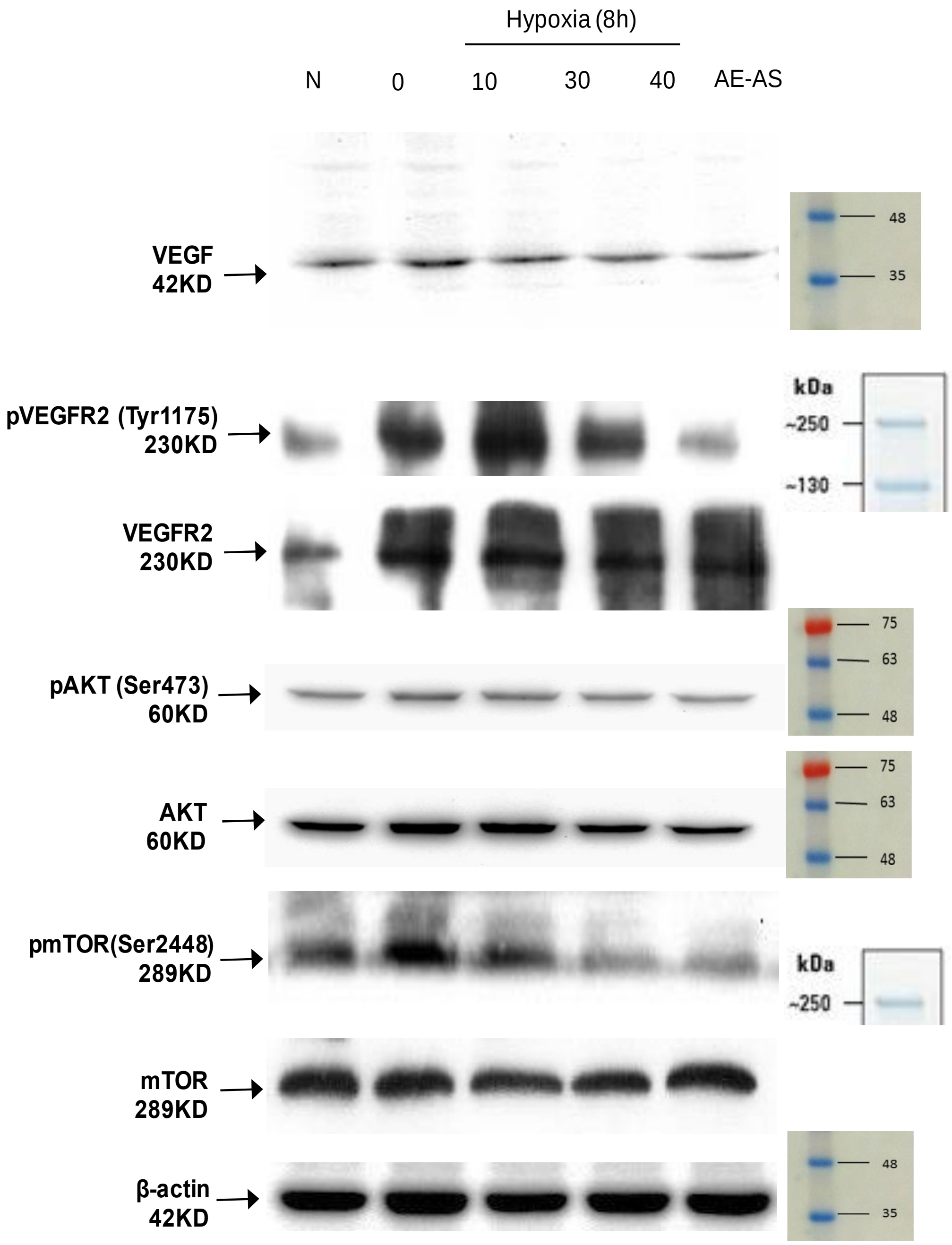

Hypoxia (8h)
N
0
10
30
40
AE-AS
VEGF
42KD
48
35
pVEGFR2 (Tyr1175)
230KD
kDa
~250
~130
VEGFR2
230KD
75
63
48
pAKT (Ser473)
60KD
75
63
48
AKT
60KD
pmTOR(Ser2448)
289KD
kDa
~250
mTOR
289KD
48
35
β-actin
42KD

Figure 2F

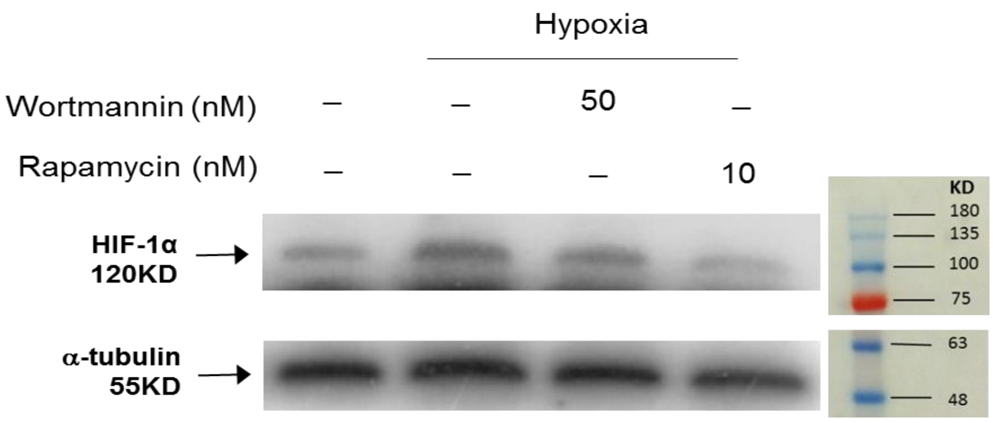

Figure 3A

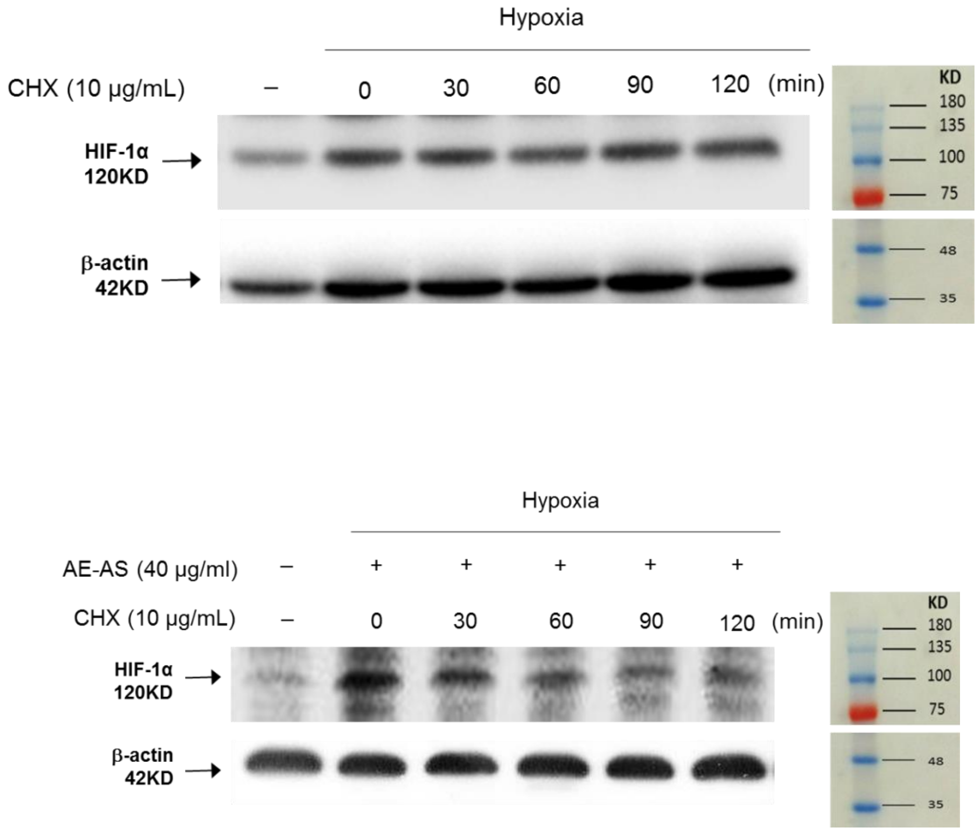

Figure 3B

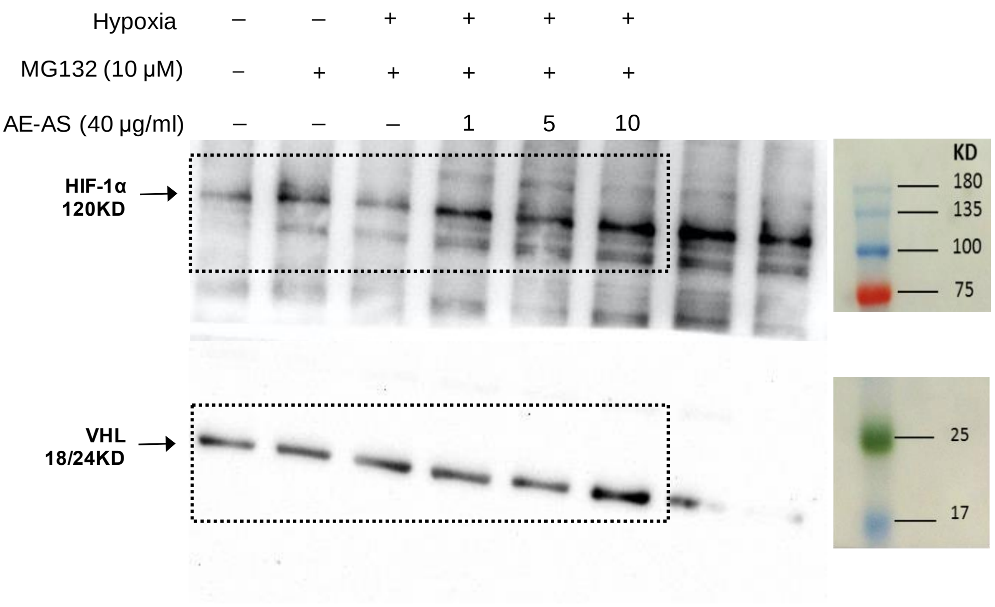

Figure 3C

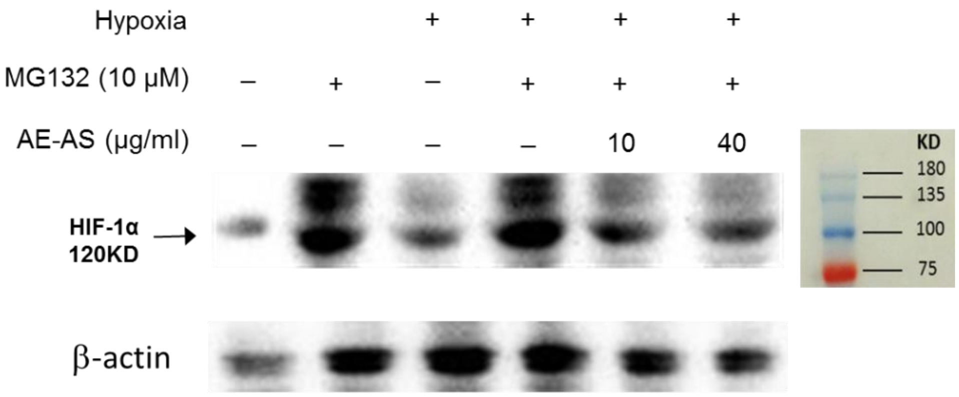

Figure 3D

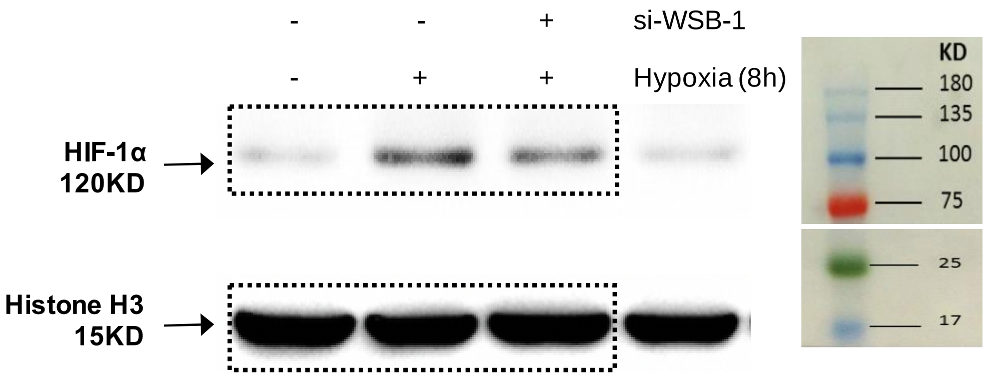

Figure 3E

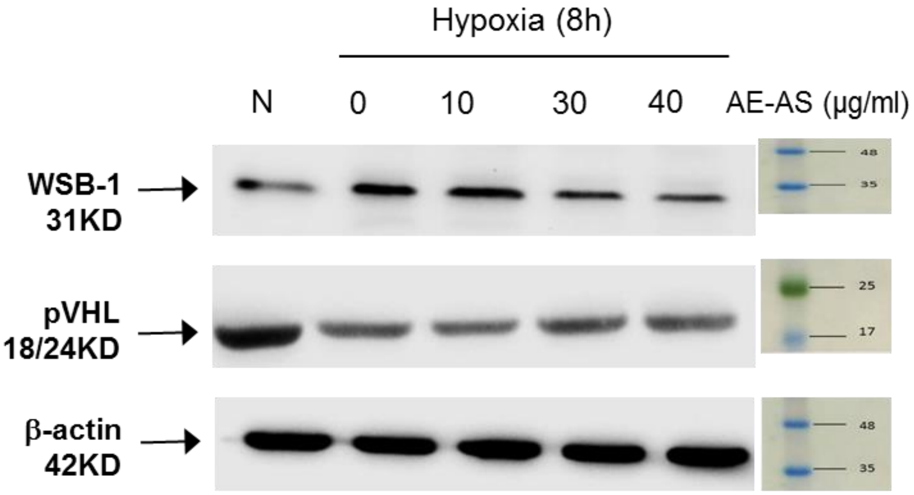

Figure 3F

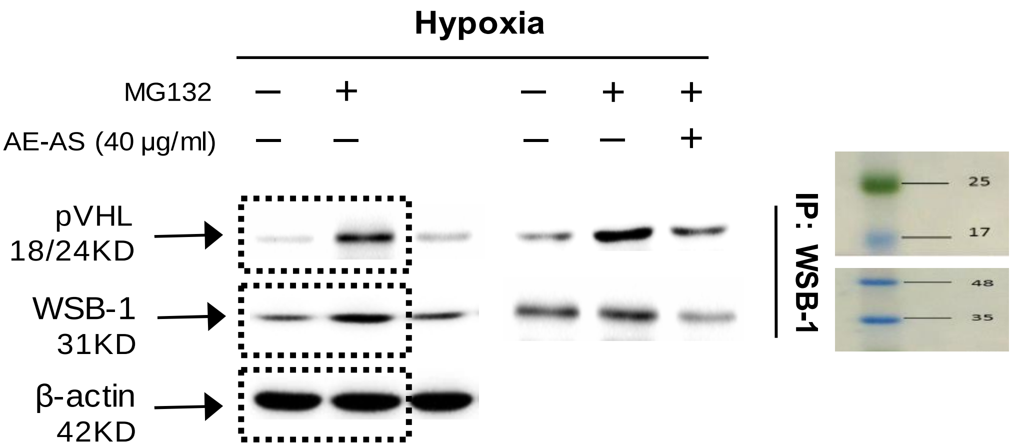

Figure 6.

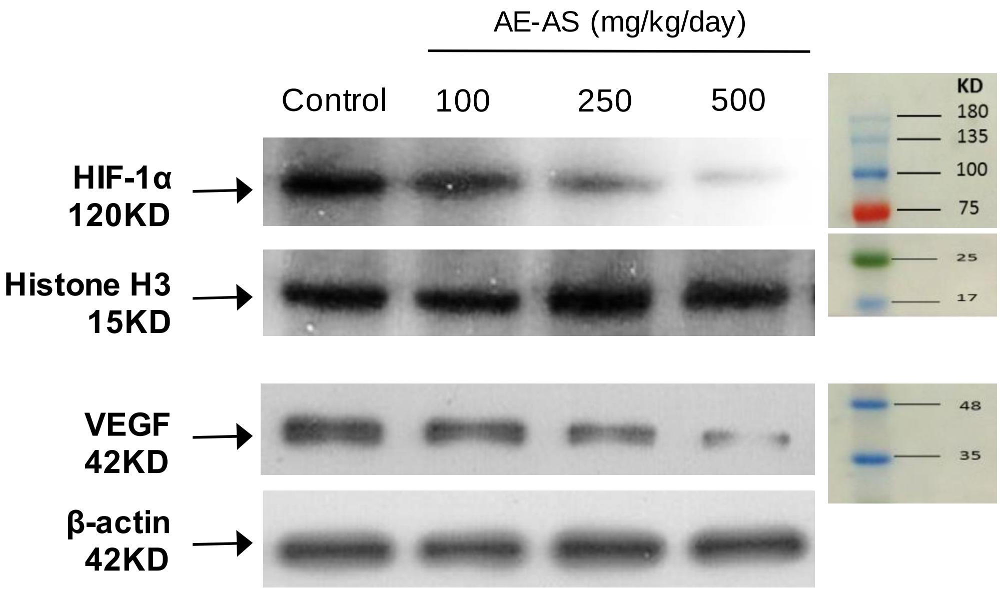

AE-AS (mg/kg/day)
Control
100
250
500
HIF-1α
120KD
Histone H3
15KD
VEGF
42KD
β-actin
42KD
KD
180
135
100
75
25
17
48
35
